# Differences of protein expression profiles, KRAS and BRAF mutation, and prognosis in right-sided colon, left-sided colon and rectal cancer

**DOI:** 10.1038/s41598-017-08413-z

**Published:** 2017-08-11

**Authors:** Xian Hua Gao, Guan Yu Yu, Hai Feng Gong, Lian Jie Liu, Yi Xu, Li Qiang Hao, Peng Liu, Zhi Hong Liu, Chen Guang Bai, Wei Zhang

**Affiliations:** 1 Department of Colorectal Surgery, Changhai Hospital, The Second Military Medical University, Shanghai, 200433 China; 2 Department of Pathology, Changhai Hospital, The Second Military Medical University, Shanghai, 200433 China

## Abstract

To compare protein expression levels, gene mutation and survival among Right-Sided Colon Cancer (RSCC), Left-Sided Colon Cancer (LSCC) and rectal cancer patients, 57 cases of RSCC, 87 LSCC and 145 rectal cancer patients were included retrospectively. Our results demonstrated significant differences existed among RSCC, LSCC and rectal cancer regarding tumor diameter, differentiation, invasion depth and TNM stage. No significant difference was identified in expression levels of MLH1, MSH2, MSH6, PMS2, β-Tubulin III, P53, Ki67 and TOPIIα, and gene mutation of KRAS and BRAF among three groups. Progression Free Survival (PFS) of RSCC was significantly lower than that of LRCC and rectal cancer. In univariate analyses, RSCC, preoperative chemoradiotherapy, poor differentiation, advanced TNM stage, elevated serum CEA and CA19-9 level, tumor deposit, perineural and vascular invasion were found to be predictive factors of shorter PFS. In multivariate analyses, only differentiation and TNM stages were found to be independent predictors of PFS. In conclusion, compared with LSCC and rectal cancer, RSCC has larger tumor size, poor differentiation, advanced TNM stage and shorter survival. The shorter survival in RSCC might be attributed to the advanced tumor stage caused by its inherent position feature of proximal colon rather than genetic difference.

## Introduction

Colorectal cancer (CRC) is the third most commonly diagnosed cancer globally, accounting for 10.0% of all new cancer cases. An estimated 746,300 of new CRC cases and 614,300 CRC deaths occurred in 2012 worldwide. It is also the fourth common cause of cancer-related deaths in men and the third in women worldwide^1^. According to the tumor position, CRCs are usually classified into three types: Right-Sided Colon Cancer (RSCC), Left-Sided Colon Cancer (LSCC) and Rectal Cancer, and each type approximately accounts for 30%^2, 3^. Colon cancers consist of RSCC and LSCC, divided at the splenic flexure. Rectal cancers are referred to lesions located within 12 cm from the anal verge. The issue whether these three types should be considered as a single entity or three distinct entities is still controversial^2^.

It is reported that rectal cancer is different from colon cancer in aetiology, genetics, anatomy, clinical manifestation, biological feature, treatment response and clinical outcomes^3–6^. Lifestyle factors such as diet, smoking and physical activity have different effects in colon cancer than in rectal cancer^7^. The treatments for rectal and colon cancer are different, depending on the TNM stage. For stage I and IV, rectal and colon cancers are commonly regarded as one entity and treated alike^3^. For stage II–III CRC, neoadjuvant radiochemotherapy is recommended for rectal cancer patients, but not for colon cancer patients. Neoadjuvant radiochemotherapy resulted in decrease of local recurrence rate, but no increase in overall survival (OS) compared to surgery alone^8^. Furthermore, a study which includes 372,130 patients from the SEER database with a median follow-up of 32 months, showed that there was no difference in OS between colon and rectal cancer^9^. Frattini’s study showed that significant differences existed in KRAS mutation and APC mutation between colon cancers and rectal cancers^2^. However, the Cancer Genome Atlas Network conducted a genome-scale analysis of 276 samples, analyzing exome sequence, DNA copy number, promoter methylation, mRNA and microRNA expression, and concluded that colon and rectal cancers had similar patterns of genomic alteration, and gene mutations of APC, TP53, SMAD4, PIK3CA and KRAS^10^. So, whether colon and rectal cancers have different gene expression and prognosis is still in debate.

The distinction between RSCC and LSCC has received increasing attention in recent years. Some suggested that they were two distinct categories of colon cancer^11^. Many publications reported that there were significant differences regarding epidemiology, clinical presentation, pathology, genetic mutations and survival between RSCC and LSCC^11^. RSCC had been reported to be older, more often female and more often poorly differentiated tumors, and have more advanced stages, increased tumor sizes and different molecular features^11–14^. Data regarding prognosis in RSCC versus LSCC are conflicting, and it remains a matter of great debate whether tumor location itself has a significant prognostic impact^12^. Most studies demonstrated a poorer survival in RSCC compared to LSCC^15–17^. In contrast, several scholars found no difference in OS between RSCC and LSCC after adjusting for various variables^11, 18, 19^. Warschkow *et al*.^12^ carried out a study including 91,416 patients, and found that RSCC patients had worse OS compared to LSCC patients; but the prognosis of RSCC was better than LSCC after matching clinical features. In addition, whether molecular features differ between LSCC and RSCC remains unclear^20^. Kuramochi *et al*.^21^ had detected mRNA expression levels of 14 signal transduction genes in 52 cases of CRC, but only identified significant differences in PTEN mRNA expression level.

Furthermore, some authors suggested that LSCC and rectal cancer shared multiple common characteristics and were different from RSCC, which was supported by several histological, genetic and methylation findings^3, 22, 23^. And a new term, Left-sided Colorectal Cancer (LCRC) which included LSCC and rectal cancer, was created^24^. There are three main types of (epi)genetic instability in CRC: (1) chromosomal instability (CIN) caused by KRAS mutations; (2) microsatellite instability (MSI) resulted from deficient DNA mismatch repair (MMR); (3) CpG island methylator phenotype (CIMP) epigenetic instability^3^. The mutational profiles (KRAS, MMR, CIMP) of LSCC and rectal cancer were similar, but were different from that of RSCC. This result was attributed to their differing origins: RSCC originated from midgut, while LCRC originated from hindgut^3^. BRAF was preferentially mutated in RSCC, and EGFR (epidermal growth factor receptor) was prevalently amplified in LCRC^3^. Class III beta-tubulin (β-Tubulin III) had been reported to express at the invasive margin of CRC, and its expression level was correlated with tumor differentiation and lymphatic metastasis^25^. The mutation incidence of p53 gene was reported to be as high as 42.4% in CRC^26^. P53 plays an important role in the transformation from colorectal normal mucosa to carcinoma through adenoma^27^. Several studies reported that gene mutation and protein expression of P53 differed significantly between RSCC and LSCRC^26, 28, 29^, but others showed that no significant association was identified between p53 protein expression and tumor site^30^. Ki67 is a marker of cell proliferation, and it plays a vital role in the development of CRC^31^. High Ki67 labeling index had been reported to be an independent prognostic biomarker in TNM III and IV CRC^32^. In addition, our previous study showed that topoisomerase II alpha (TOPIIα) expression was related with T stage, N stage, recurrence and prognosis^33^.

In the light of the aforementioned considerations, it is urgent for us to explore the possible differences of gene expression level and prognosis among RSCC, LSCC and rectal cancers. Knowledge of the molecular differences would help us to improve the diagnosis and treatment strategy in clinical practice. Thus, we investigated the protein expression levels of MLH1, MSH2, MSH6, PMS2, β-Tubulin III, P53, Ki67 and TOPIIα, and the gene mutation of KRAS and BRAF in 289 specimens of sporadic CRC patients, and their implications on survival were also investigated.

## Methods

### Patients

From January 2015 to December 2016, 289 cases of sporadic CRC patients were recruited from the Department of Colorectal Surgery of Changhai Hospital, Second Military Medical University, Shanghai, China. All patients received radical resection of the primary tumor. The clinicopathological characteristics were extracted from the electronic medical records. All patients were followed up every 3 months, with a median follow up period of 10 months, ranging from 3 to 23 months. Informed consent had been obtained from all patients and the project had been approved by the Ethics Committee of Changhai Hospital, Second Military Medical University. All methods were performed in accordance with the relevant guidelines and regulations.

### Immunohistochemistry

Paraffin-embedded tumor tissues were examined for MLH1, MSH2, MSH6, PMS2, β-tubulin III, P53, Ki67 and TopIIα expression, using the Envision method following the manufacturer’s instruction. After deparaffinization and re-hydration, antigen retrieval was done with Citrate buffer (0.01 mol/L, pH = 6.0) by pressure cooker. Primary antibodies (Table 1) were incubated on the slides for 2 hours at room temperature in a hydrated chamber. The sections were stained with DAB and counterstained with Mayer’s hematoxylin, washed again, dehydrated in alcohol, cleared in xylene, mounted with Pertex mounting medium, and coverslipped. All sections were scored blindly by two pathologists (Xu Y & Bai CG) under microscope, by randomly selecting 10 high-power (×400) view fields in each sample and scoring the protein expression in tumor cells.

**Table 1:** The primary antibody of MLH1, MSH2, MSH6, PMS2, β-Tubulin III, P53, Ki67 and TOPIIα used in this study.

Antibody	Corporation	Manufacturer City	Product code	Dilution	Clonality
MLH1	Maixin Biotech	Fuzhou, China	MAB-0642	1:50	G168-15
MSH2	Maixin Biotech	Fuzhou, China	MAB-0291	1:50	25D12
MSH6	Maixin Biotech	Fuzhou, China	MAB-0643	1:50	44
PMS2	Maixin Biotech	Fuzhou, China	MAB-0656	1:50	MOR4G
β-Tubulin III	Maixin Biotech	Fuzhou, China	MAB-0636	1:100	TUJ1
P53	Maixin Biotech	Fuzhou, China	Kit-0010	1:100	Do-7
Ki-67	Maixin Biotech	Fuzhou, China	Kit-0005	1:100	MIB-1
TOPIIα	Maixin Biotech	Fuzhou, China	MAB-0588	1:100	3F6

Expression defects of MLH1, MSH2, MSH6 and PMS2 proteins were defined as complete absence of detectable nuclear staining in tumor cells. Intact nuclear staining of the colorectal crypts of the peritumoral normal mucosa, stromal cells and lymphocytes served as internal positive control and was required for adequate evaluation^34^.

To measure the expression level of β-tubulin III, P53, Ki67 and TopIIα, each sample was scored according to the intensity of the nucleic or cytoplasmic staining (0, no staining; 1, weak staining; 2, moderate staining; and 3, strong staining) and the extent of stained cells (0%, 0; 1–25%, 1; 26–50%, 2; 51–75%, 3; and 76–100%, 4). The multiplication of the intensity and extent score was used as the final staining scores (0 to 12). Tumors having final staining scores of 0, 1~4, 5~8 and 9~12 were considered to be negative (−), slightly positive (+), moderately positive (++) and strongly positive (+++), respectively^35^. There was a close agreement on staining intensity (91%) and staining extent (93%) between the two pathologists. Disagreements were resolved by consensus. Immunohistochemical labeling index was defined as the percentage of positive nuclei in relation to the whole tumor area.

### Sample preparation, DNA extraction, amplification of *KRAS* and *BRAF* genes

Surgically resected primary CRC tumor tissue specimens were fixed in formalin and preserved in paraffin blocks for histological examination. After evaluating the standard hematoxylin/eosin-stained slides from each specimen, appropriate samples were specifically chosen by a pathologist to include predominantly tumor cells without significant necrosis or inflammation^36^. Eight 10 μm-thick formalin-fixed paraffin-embedded (FFPE) sections were used for this study, and placed in 2-mL sterile Eppendorf tubes. AmoyDx FFPE DNA Kit (AmoyDx, Xiamen, China) was used for DNA extraction. Human genomic DNA was amplified for KRAS in exons 2, 3 and 4 (codons 12, 13, 59, 61, 117 and 146), and BRAF in exon 15 (codon 600) by using AmoyDx gene mutation real-time PCR kits (AmoyDx, Xiamen, China). All the experiments are performed following manufacturer’s instructions. 5 μL DNA was used for PCR amplification in each reaction, the PCR cycling conditions were: 5 min incubation at 95 °C, followed by 15 cycles of 95 °C for 25 sec, 64 °C for 20 sec, 72 °C for 20 sec and then 31 cycles of 93 °C for 25 sec, 60 °C for 35 sec, 72 °C for 20 sec. Fluorescent signal was collected from FAM and HEX channels. Each PCR run must contain one negative control and one positive control. KRAS and BRAF mutation status were determined according to the Ct value as indicated in the manufacturer’s instructions.

### Statistical analysis

Associations between tumor position and categorical clinicopathological variables were analyzed by χ^2^ test, using Fisher Exact test if one or more expects in the cross table is less than 5. Numerical data which were consistent with normal distribution were presented as the mean ± standard deviation, and comparisons were performed with the student’s t-test or one-way ANOVA analysis. Numerical data which were inconsistent with normal distribution were presented as median (minimum-maximum), and comparisons were performed with nonparametric test. The Progression Free Survival (PFS) was estimated by the Kaplan-Meier method and survival differences were analyzed with the log-rank test. The Cox proportional hazards model was used for multivariate analysis of prognostic factors. *P* < 0.05 was considered statistically significant (two-sided). All statistical analyses were conducted using SPSS 17.0 statistical software (SPSS, Inc., Chicago, IL, USA).

## Results

A total of 289 cases of CRC patients were included, consisting of 57 cases of RSCC, 87 LSCC and 145 rectal cancers (Table 2). Of the 289 CRC patients, 186 were males and 103 were females, with a mean age of 59.6 years. Thirty-three (33/289, 11.4%) patients received preoperative chemoradiotherapy, 156 (156/289, 54.0%) received postoperative chemoradiotherapy; 82 were resected laparoscopically, and the remaining 207 were removed by open surgery. All of the primary tumors were resected radically, and combined resections of metastatic lesions were performed in 29 patients. There were 60, 82, 89 and 58 cases of stage I, II, III and IV according to the UICC-AJCC TNM stage classification system (7th edition).

**Table 2:** Relationship between Tumor Position and Clinicopathological Parameters in 289 Colorectal Cancer Patients.

Clinicopathological Parameters	RSCC^#^ (n = 57)	LSCC▴ (n = 87)	Rectal cancer (n = 145)	Total (n = 289)	P1*	P2*	P3*	P4*	P5*
Gender					0.869	0.615	0.648	0.915	0.868
Male	35	57	94	186					
Female	22	30	51	103					
Age (year)	58.6 ± 12.9	60.0 ± 11.7	59.8 ± 10.1	59.6 ± 11.2	0.743	0.511	0.484	0.910	0.775
Preoperative chemotherapy					0.845	0.949	0.691	0.591	0.565
No	50	76	130	256					
Yes	7	11	15	33					
Open or Laparoscopic					**0.004**	**0.003**	**0.001**	0.885	**0.073**
Open	51	59	97	207					
Laparoscopic	6	28	48	82					
Gross Type					0.598	0.567	0.318	0.648	0.394
Protruding	9	17	32	58					
Ulcerative & Infiltratie	48	70	113	231					
Diameter (cm)	5.4 ± 2.3	4.3 ± 1.5	4.1 ± 1.6	4.4 ± 1.8	**0.000**	**0.002**	**0.000**	0.415	**0.004**
Differentiation					**0.000**	**0.000**	**0.000**	0.363	0.066
Well & Moderate	33	78	124	235					
Poor☆	24	9	21	54					
T					**0.000**	0.110	**0.000**	**0.015**	**0.000**
T1-T2	5	16	48	69					
T3-T4	52	71	97	220					
N					0.708	0.437	0.466	0.891	0.772
N0	29	50	82	161					
N1-N2	28	37	63	128					
M					0.667	0.980	0.502	0.423	0.368
M0	44	67	118	229					
M1	13	20	27	60					
TNM					**0.011**	0.711	**0.008**	**0.042**	**0.001**
1	5	12	43	60					
2	22	27	33	82					
3	17	28	44	89					
4	13	20	25	58					
CEA (ng/mL)					0.590	0.951	0.473	0.273	0.259
Unknown	3	5	3	11					
<5	31	45	84	160					
>=5	23	37	58	118					
CA199 (U/mL)					0.298	0.439	0.142	0.297	0.200
Unknown	4	5	3	12					
<37	40	69	116	225					
>=37	13	13	26	52					
Tumor Deposit					0.207	0.677	0.311	0.094	0.083
No	47	74	110	231					
Yes	10	13	35	58					
Perineural Invasion					0.643	0.967	0.505	0.410	0.348
No	49	75	119	243					
Yes	8	12	26	46					
Vascular Invasion					0.454	0.493	0.757	0.213	0.305
No	49	71	127	247					
Yes	8	16	18	42					

^#^RSCC: Right-sided colon cancer; ▴LSCC: Left-sided colon cancer; *P1: comparison of three group; P2: RSCC vs. LSCC; P3: RSCC vs. rectal; P4: LSCC vs. rectal; P5:colon vs. rectal; ☆Poorly differentiated, Mucinous adenocarcinoma, signet ring cell carcinoma.

### Comparisons of clinicopathological parameters among RSCC, LSCC and rectal cancer patients

As demonstrated in Table 2, significant differences were observed among the RSCC, LSCC and rectal cancer patients regarding surgical procedure (Open/Laparoscopic), tumor diameter, differentiation, invasion depth (T) and TNM stage (all P < 0.05). No significant difference was observed in other characteristics, including gender, age, preoperative chemoradiotherapy, gross type, lymph node metastasis(N), distant metastasis(M), serum CEA level, serum CA 19-9 level, tumor deposit, perineural invasion and vascular invasion (Table 2, all P > 0.05). Of the 58 cases of TNM stage IV patients, 38 had received postoperative chemoradiotherapy, 12 had received postoperative biological therapy, and 8 had received second line chemoradiotherapy after recurrence. No significant difference was identified in postoperative chemoradiotherapy, biological therapy and second line chemoradiotherapy among the three groups (Table 3, all P > 0.05).

**Table 3:** Postoperative chemoradiotherapy and Biological therapy in TNM stage IV Colorectal Cancer Patients.

Postoperative treatment	Right-sided colon cancer (n = 13)	Left-sided colon cancer (n = 20)	Rectal cancer (n = 25)	Total (n = 58)	P
Postoperative chemoradiotherapy					0.386
No	3	1	7	11	
Yes	8	16	14	38	
Unknown	2	3	4	9	
Postoperative biological therapy					0.551
No	9	8	15	32	
Anti-EGFR	1	4	2	7	
Anti-VEGF	1	3	1	5	
Unknown	2	5	7	14	
Postoperative second line chemoradiotherapy					0.089
No	7	13	21	41	
Yes	4	4	0	8	
Unknown	2	3	4	9	

### Comparisons of clinicopathological parameters between each two groups

Compared with LSCC, RSCC was associated with less laparoscopic resection (P = 0.003), larger tumor size (P = 0.002) and poor differentiation (P < 0.001) (Table 2). Compared with rectal cancer, RSCC was associated with less laparoscopic resection (P = 0.001), larger tumor size (P < 0.001), poor differentiation (P < 0.001), advanced T stage (P < 0.001) and advanced TNM stage (P = 0.008) (Table 2). Compared with rectal cancer, LSCC was associated with advanced T stage (P = 0.015) and advanced TNM stage (P = 0.042) (Table 2). Compared with rectal cancer, colon cancer was associated with larger tumor size (P = 0.004), advanced T stage (P < 0.001) and advanced TNM stage (P = 0.001) (Table 2).

### Comparisons of protein expression levels, KRAS and BRAF mutation among RSCC, LSCC and rectal cancer patients

**Figure 1:**
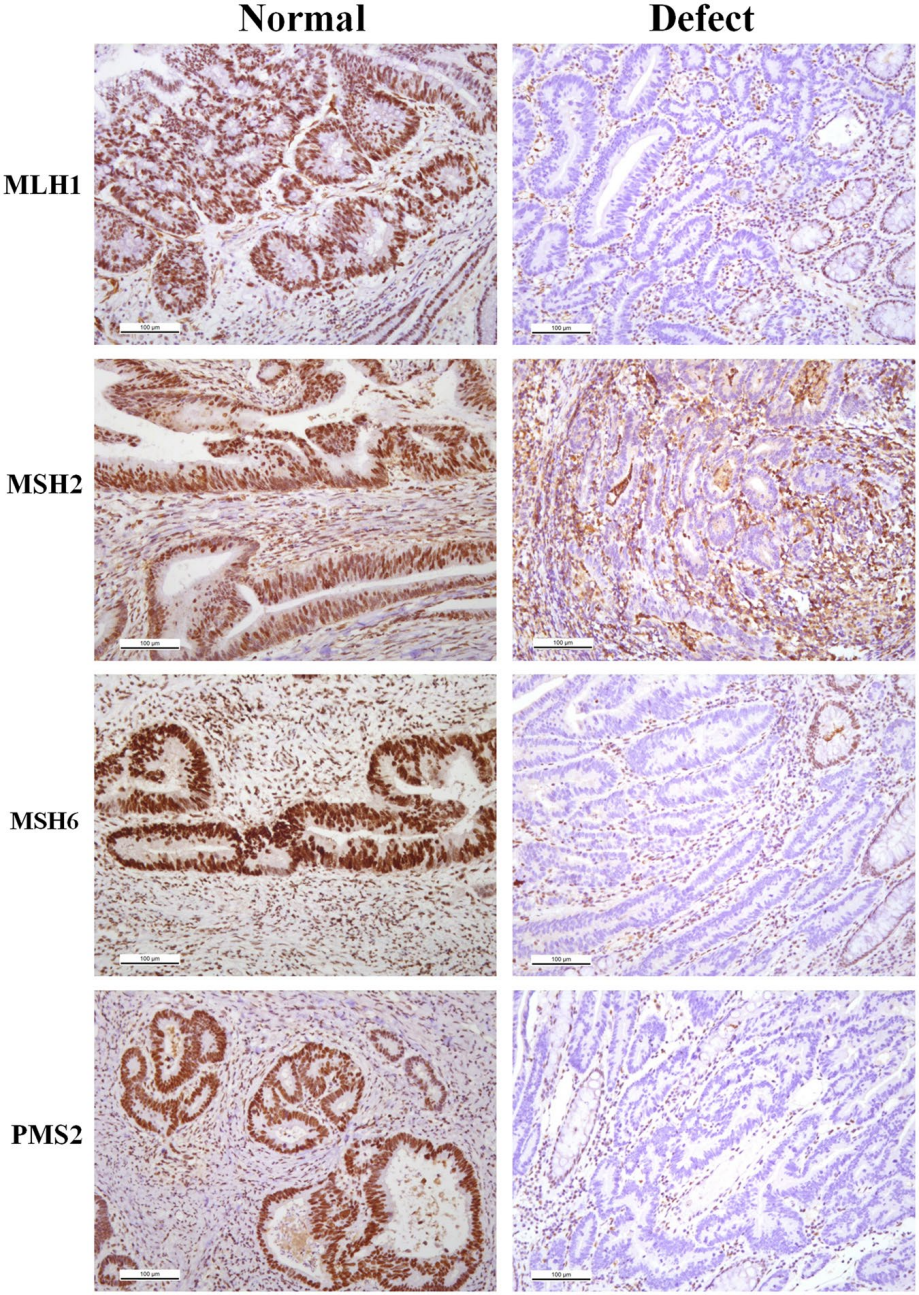
Representative images of MLH1, MSH2, MSH6 and PMS2 in colorectal cancer tissue.

**Figure 2:**
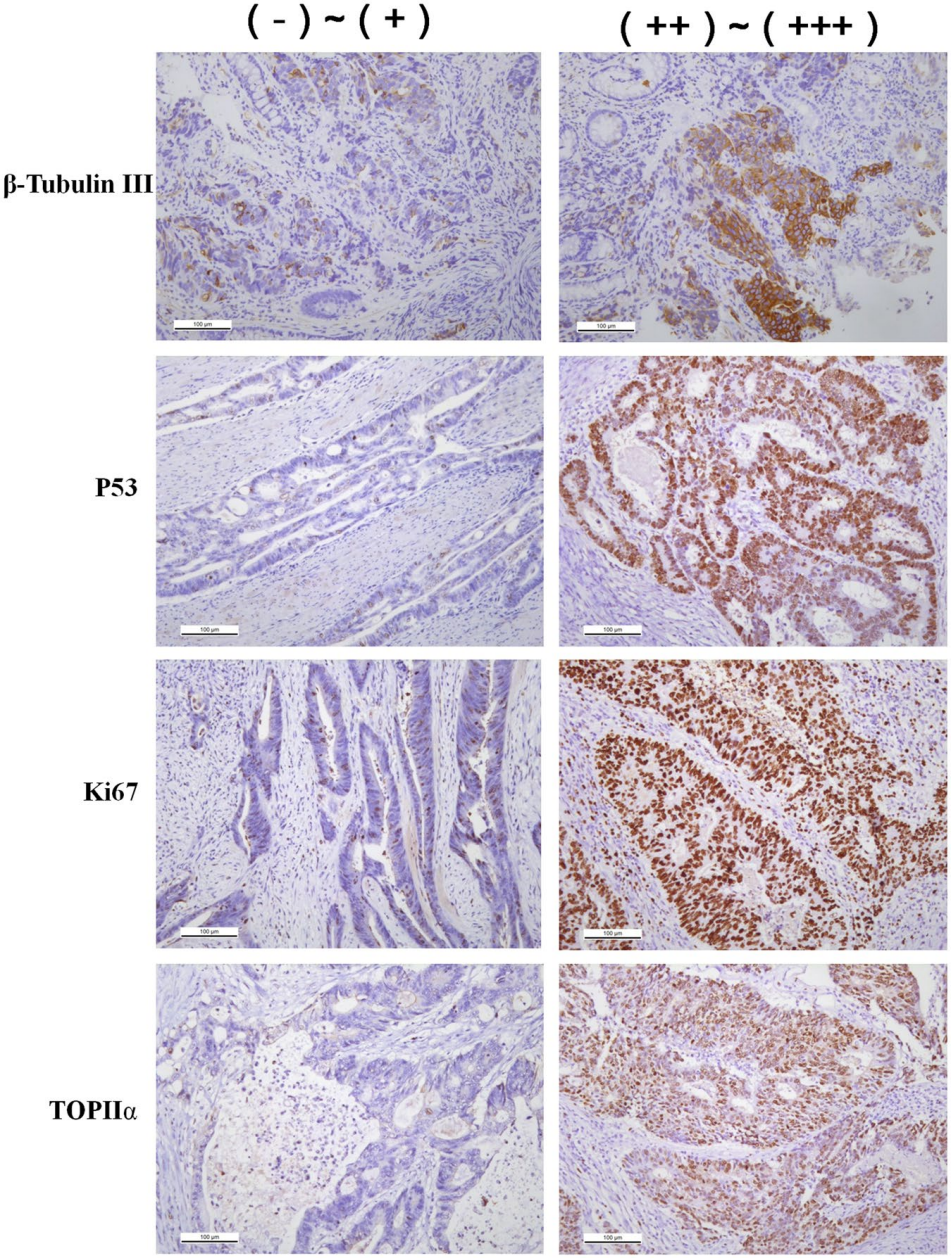
Representative images of different expression level of β-Tubublin III, P53, Ki67 and TOPIIα in colorectal cancer (×200).

**Figure 3:**
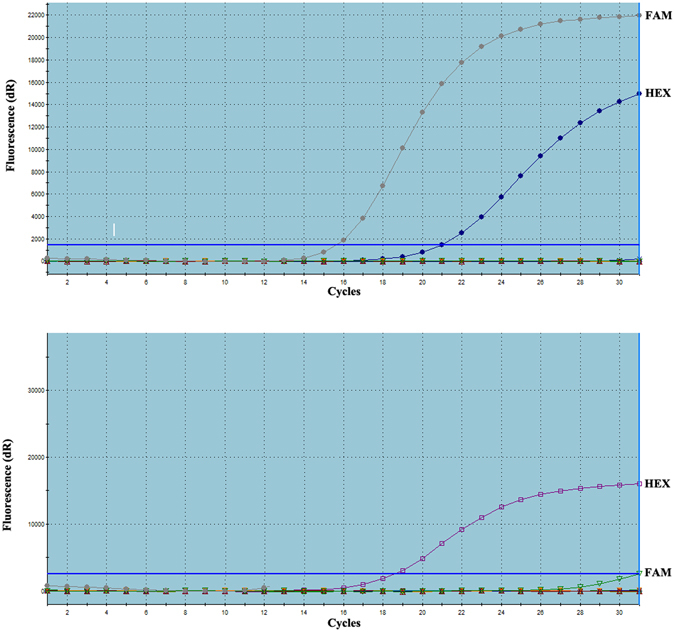
Graphic representations of gene mutation detection of KRAS (A, FAM(+)HEX(+), mutant type) and BRAF (B, FAM(−)HEX(+), wild type). (HEX: Internal reference sample, FAM: Test sample).

No significant difference was observed among RSCC, LSCC and rectal cancer, regarding protein expression levels of MLH1, MSH2, MSH6, PMS2, β-tubulin III, P53, Ki67 and TopIIα (Figs 1 and 2 & Table 4). Similarly, no significant difference was identified in gene mutation incidences of KRAS and BRAF (Fig. 3, Table 4). Immunohistochemical labeling index of TOPIIα in RSCC was significantly lower than that in LSCC (P = 0.007) and that in rectal cancer (P = 0.010) (Table 4).

**Table 4:** Relationship between Tumor Position and Gene Mutation, Protein Expression Levels in 289 Colorectal Cancer Patients.

Gene Mutation & Expression Level	Right-sided colon cancer (n = 57)	Left-sided colon cancer (n = 87)	Rectal cancer (n = 145)	Total (n = 289)	P1*	P2*	P3*	P4*	P5*
KRAS					0.685	0.637	0.392	0.673	0.464
Wild	27	42	59	128					
Mutant	16	30	48	94					
Unknown	14	15	38	67					
BRAF					0.074	0.144	0.068	0.769	0.202
Wild	39	70	106	215					
Mutant	3	1	1	5					
Unknown	15	16	38	69					
MLH1					0.735	0.614	0.860	0.509	0.701
Defect	6	13	17	36					
Normal	44	67	114	225					
Unknown☆	7	7	14	28					
MSH2					0.898	0.641	0.786	0.792	0.974
Defect	2	2	4	8					
Normal	50	80	127	257					
Unknown☆	5	5	14	24					
MSH6					0.989	0.886	0.945	0.920	0.972
Defect	4	6	10	20					
Normal	46	76	120	242					
Unknown☆	7	5	15	27					
PMS2					0.497	0.388	0.828	0.268	0.566
Defect	1	0	2	3					
Normal	49	79	128	256					
Unknown☆	7	8	15	30					
β-Tubulin III					0.452	0.280	0.221	0.957	0.563
(−)~(+)	8	27	52	87					
(++)~(+++)	5	8	15	28					
Unknown☆	44	52	78	174					
TOPIIα					0.223	0.088	0.317	0.308	0.818
(−)~(+)	41	54	96	191					
(++)~(+++)	11	29	38	78					
Unknown☆	5	4	11	20					
P53					0.660	0.366	0.607	0.585	0.902
(−)~(+)	26	36	63	125					
(++)~(+++)	24	46	69	139					
Unknown☆	7	5	13	25					
Ki67					0.875	0.922	0.746	0.619	0.612
(−)~(+)	3	5	6	14					
(++)~(+++)	51	79	129	259					
Unknown☆	3	3	10	16					
TOPIIα index (%)	19 (0–70)	27 (0–90)	25 (0–90)	20 (0–90)	**0.015**	**0.007**	**0.010**	0.635	0.322
P53 index (%)	38 (0–95)	46 (0–95)	42 (0–95)	45 (0–95)	0.311	0.138	0.430	0.323	0.765
Ki67 index (%)	68 (5–90)	69 (0–100)	69 (10–90)	70 (0–100)	0.651	0.439	0.878	0.412	0.622

^#^RSCC: Right-sided colon cancer; ▴LSCC: Left-sided colon cancer; *P1: comparison of three group; P2: RSCC vs. LSCC; P3: RSCC vs. rectal; P4: LSCC vs. rectal; P5:colon vs. rectal; ☆All of the “Unknown” groups were not taken into analysis in this table.

### Influencing factors of Progression Free Survival (PFS)

**Figure 4:**
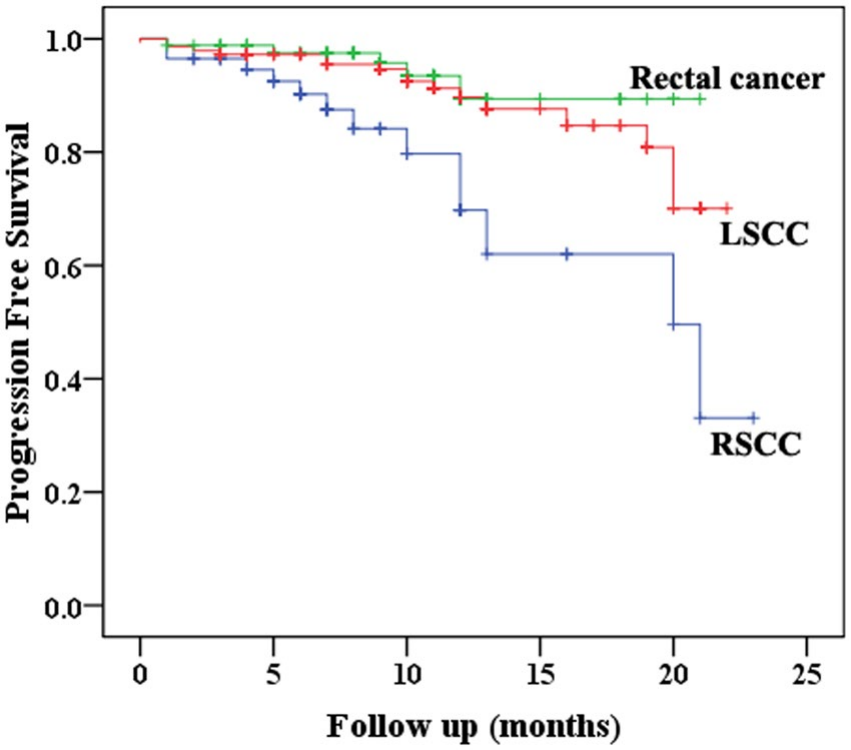
The relationship between tumor location and Progression Free Survival (PFS, P = 0.002) in 289 cases of Stage I~IV colorectal cancer patients (RSCC: Right-Sided Colon Cancer; LSCC: Left-Sided Colon Cancer).

Our results showed that tumor location was related with PFS (Fig. 4, P = 0.002). The PFS of RSCC was significantly shorter than that of LRCC (P = 0.003) and rectal cancer (P = 0.004). No significant difference was identified in PFS between LSCC and rectal cancer patients (P = 0.392).

By univariate analysis, RSCC (P = 0.018), preoperative chemoradiotherapy (P = 0.010), poor differentiation (P = 0.001), advanced T stage (P = 0.018), lymph node metastasis (P = 0.001), distant metastasis (P < 0.001), advanced TNM stage (P < 0.001), elevated serum CEA level (P = 0.035), elevated serum CA19-9 level (P = 0.025), tumor deposit (P = 0.001), perineural invasion (P = 0.003) and vascular invasion (P = 0.001) were all found to be associated with shorter PFS (Table 5). Multivariate analyses only identified poor differentiation (P = 0.048) and advanced TNM stage (P < 0.001) as predictor of shorter PFS (Table 6).

**Table 5:** Univariate Analyses of Factors Associated With Progression Free Survival (n = 289).

Variables	95%CI	P
Tumor Position (Right vs. Left&Rectum)^#^	0.612 (0.407–0.919)	**0.018**
Gender (Male vs. Female)	1.428 (0.720–2.833)	0.308
Age (<65 y vs. >=65 y)	1.098 (0.549–2.196)	0.791
Preoperative chemotherapy (No vs. Yes)	3.295 (1.332–8.153)	**0.010**
Preoperative radiation (No vs. Yes)	0.048 (0–1858.733)	0.573
Combined Resection (No vs. Yes)	2.087 (0.863–5.046)	0.102
Open or Laparoscopic (Open vs. Laparoscopic)	0.611 (0.275–1.357)	0.226
Gross Type (Protruding vs. (Ulcerative, Infiltratie))	1.919 (0.581–6.342)	0.285
Circumference (<1/2 vs. >=1/2)	1.037 (0.925–1.162)	0.538
Differentiation (Well&Moderate vs. Poor)	3.161 (1.560–6.407)	**0.001**
T stage ((T1-T2) vs. (T3-T4))	5.586 (1.338–23.317)	**0.018**
N (N0 vs. (N1-N2))	3.586 (1.673–7.686)	**0.001**
M (M0 vs. M1)	4.356 (2.211–8.579)	**0.000**
TNM ((1–2) vs. (3–4))	2.673 (1.775–4.026)	**0.000**
CEA (<5ng/mL vs. >=5ng/mL)	2.007 (1.051–3.833)	**0.035**
CA199 (<37 U/mL vs. >=37 U/mL)	2.225 (1.106–4.473)	**0.025**
KRAS (Wild vs. Mutant)	0.945 (0.842–1.061)	0.340
BRAF (Wild vs. Mutant)	0.954 (0.856–1.064)	0.397
Tumor Deposit (No vs. Yes)	3.270 (1.659–6.445)	**0.001**
Perineural Invasion (No vs. Yes)	2.862 (1.414–5.789)	**0.003**
Vascular Invasion (No vs. Yes)	3.166 (1.556–6.444)	**0.001**
MLH1 (Defect vs. Normal)	0.966 (0.837–1.114)	0.619
MSH2 (Defect vs. Normal)	0.934 (0.785–1.111)	0.438
MSH6 (Defect vs. Normal)	0.905 (0.760–1.078)	0.262
PMS2 (Defect vs. Normal)	0.907 (0.755–1.090)	0.298
β-Tubulin III ((−)~(+) vs. (++)~(+++))	1.016 (0.928–1.111)	0.735
TOPIIα ((−)~(+) vs. (++)~(+++))	0.930 (0.748–1.158)	0.518
P53 ((−)~(+) vs. (++)~(+++))	0.938 (0.774–1.137)	0.514
Ki67 ((−)~(+) vs. (++)~(+++))	1.001 (0.811–1.237)	0.991

^#^Right-sided colon cancer vs. Left -sided colon cancer & Rectal cancer.

**Table 6:** Multivariate Analyses of Factors Associated With Progression Free Survival (n = 289).

Variables	95%CI	P
Tumor Position (Right vs. Left &Rectum)^#^	0.646 (0.415–1.006)	0.053
Preoperative chemotherapy (No vs. Yes)	1.881 (0.553–6.397)	0.312
Differentiation (Well& Moderate vs. Poor)	2.066 (1.005–4.247)	**0.048**
T stage ((T1-T2) vs. (T3-T4))	0.622 (0.105–3.692)	0.601
N (N0 vs. (N1-N2))	0.470 (0.124–1.789)	0.268
M (M0 vs. M1)	0.287 (0.052–1.591)	0.153
TNM ((1–2) vs. (3–4))	2.484 (1.640–3.763)	**0.000**
CEA (<5 ng/mL vs. >=5 ng/mL)	1.709 (0.846–3.450)	0.135
CA199 (<37 U/mL vs. >=37 U/mL)	1.183 (0.552–2.535)	0.665
Tumor Deposit (No vs. Yes)	2.098 (0.870–5.058)	0.099
Perineural Invasion (No vs. Yes)	1.484 (0.700–3.144)	0.303
Vascular Invasion (No vs. Yes)	1.474 (0.653–3.331)	0.351

^#^Right-sided colon cancer vs. Left -sided colon cancer & Rectal cancer.

## Discussion

The difference among RSCC, LSCC and rectal cancer patients has been remaining a serious debate for a long time. Our comparative study showed that these three groups had similar baseline in most clinicopathological characteristics. But there were still significant differences in surgical procedure (Open/Laparoscopic), tumor diameter, differentiation, invasion depth (T) and TNM stage among the three groups. Compared with LCRC, RSCC had less laparoscopic resection, larger tumor size and poor differentiation. As far as T stage and TNM stage were concerned, RSCC was also found to have similar tumor stage compared with LSCC in our study, which might have something to do with the small sample size. Our results were consistent with that in the published literature, which reported that RSCC had more advanced tumor stages, increased tumor sizes and poorly differentiation^3, 11–14^. Due to the larger bowel lumen, RSCC usually becomes symptomatic later than LSCC, which in turn leads to later diagnosis, larger tumor size and advanced tumor stage^37, 38^. Secondly, RSCC is located far away from the anal verge, so it is more difficult to be discovered by digit rectal examination and sigmoidoscopy. Hugen *et al*.^39^ reported that the frequency of mucinous and signet-ring cell tumors was higher in RSCC (45%) than in that in LCRC (20%). It was consistent with our results, but the underlying cause for poorer differentiation in RSCC is still unknown. Some oncologists hypothesized that it could be attributed to different underlying genetic and biological features^20, 40^. The reported differences in age, gender, perineural invasion and vascular invasion by other authors^37^ was not found in our study, which may be attributed to the relatively small sample size. In our study, RSCC was less likely to undergo laparoscopic resection, which may have something to do with the relatively higher difficulty of laparoscopic right hemicolectomy.

It had been hypothesized that there were significant differences in molecular features between RSCC and LSCC, which might serve as the cause of clinicopathological differences^40^. RSCC was reported to have a higher frequency of KRAS mutation than LSCC (57.3% vs 40.4%; P < 0.0001)^41^, and a higher incidence of BRAF mutation with 18.4–22.4% in RSCC and 1.3–7.8% in LCRC^42^. But RSCC had also been reported to be associated with more mutant KRAS and more wild-type BRAF tumors^19^. But no significant difference was found in KRAS and BRAF mutation in our study. Similarly, except for the TOPIIα immunostaining index, no significant difference was found in protein expression levels of MLH1, MSH2, MSH6, PMS2, β-tubulin III, P53, Ki67 and TopIIα between RSCC and LSCC. Our results were consistent with that of Zhu *et al*.^20^ and Cancer Genome Atlas Network^43^. Zhu *et al*. compared gene expression profiling of RSCC and LSCC using the Human Genome Array gene chip in 100 cases of patients, but only 11 genes were identified to be differentially expressed between RSCC and LSCC^20^. Cancer Genome Atlas Network conducted a genome-scale analysis of 276 samples, analyzing exome sequence, DNA copy number, promoter methylation, mRNA and microRNA expression, and concluded colon and rectal cancers had similar patterns of genomic alteration^43^. No significant relationship was found between p53 protein expression and tumor position in our study, which was consistent with the results of Ghavam-Nasiri^30^. The reported prognostic role of P53, Ki67 and TopIIα expression in the literature is conflicting^44–46^. It confirmed our results, which showed none of β-tubulin III, P53, Ki67 and TopIIα expression level was not prognostic factor in CRC.

Our study demonstrated that RSCC patients had shorter PFS compared with LCRC patient. Our result was consistent with that in most literature. Petrelli *et al*.^47^ conducted a systematic review including 66 studies and 1,437,846 patients, and found that RSCC had shorter OS than LSCC. And Lee’s systematic review showed that RSCC had shorter OS than LCRC^24^. He also suggested that CRC should be classified into two types: the RSCC and the LCRC^24^.

Since the follow-up is relatively short and few patients died in this period, so it is unreasonable for us to compare OS. In addition, since combined resections of metastatic lesions were performed in only 29 of the 60 stage IV patients with distant metastasis, so the Disease Free Survival (DFS) can’t be applied in the other 31 patients with residual tumor. For these reasons, we chose PFS to measure survival in our study. We proved that RSCC patients had longer PFS than LCRC patients, but no difference between LSCC and rectal cancer. In univariate analysis, tumor position (RSCC vs. LSCC&Rectal), preoperative chemoradiotherapy, poor differentiation, TNM stage, serum CEA and CA19-9 level, tumor deposit, perineural and vascular invasion were all found to be predictor of PFS. Petrelli *et al*. summarized that TNM stage, differentiation and vascular invasion were well-recognized risk factors for CRC^47^. Mutation of KRAS and BRAF had also been reported to predict poor survival in CRC patients^13, 48, 49^, but no similar finding was identified in our study which may be related with relatively small sample size and short follow up period. NRAS mutations (codons 12, 13 and 61) were reported to occur in 3–5% of colorectal cancer^48, 49^. NRAS mutation was a predictive factor of response to anti-EGFR biological therapy and OS^49^. In our study, only 9 of the included 289 colorectal cancer patients had undergone the NRAS tests. It is difficult to reach statistical significance with such a small sample size, so NRAS gene mutation status was not analyzed in this study. A meta-analysis of FIRE-3, SWOG 80405 and PEAK trials indicated that RAS wild LSCC patients benefited more from anti-EGFR treatment (P < 0.001), while RSCC patients benefit more from anti-VEGF treatment (P > 0.05)^50^. Since no significant difference was identified in postoperative biological therapy in our study, it might not be a major contributing factor to survivals of our patients.

In the following multivariate analysis, only differentiation and TNM stage were found to be independent predictor of PFS. It indicated that tumor position might not be an independent influencing factor of PFS. Many authors also found that no difference existed in survival between RSCC and LSCC after adjusting for various variables^11, 18, 19^. In terms of these phenomena, we suggest the survival difference between RSCC and LCRC would be caused by differences in tumor stage. The RSCC’s larger lumen and longer distance from anal verge makes it less symptomatic and difficult to be discovered, which then leads to advanced tumor stage and poor prognosis^38^.

In conclusion, RSCC patients have larger tumor size, poor differentiation, advanced TNM stage and poor survival, compared with LCRC patients. Except for TOPIIα immunostaining index, no significant difference was identified in the expression levels of MLH1, MSH2, MSH6, PMS2, β-tubulin III, P53, Ki67 and TopIIα, and gene mutation of KRAS and BRAF. The shorter survival in RSCC might be attributed to the advanced tumor stage caused by its inherent position feature of proximal colon.
